# Gender gap at a large European urological congress: still at the beginning

**DOI:** 10.1007/s00345-021-03777-4

**Published:** 2021-07-04

**Authors:** Tanja Hüsch, Axel Haferkamp, Christian Thomas, Joachim Steffens, Paolo Fornara, Jennifer Kranz

**Affiliations:** 1 Department of Urology and Pediatric Urology, University Medical Center of Johannes Gutenberg University, Langenbeckstr. 1, 55131 Mainz, Germany; 2 Department of Urology, University Hospital Dresden, Dresden, Germany; 3 Department of Urology and Pediatric Urology, St. Antonius-Hospital gGmbH, Eschweiler, Germany; 4 Department of Urology and Kidney Transplantation, Martin-Luther-University, Halle (Saale), Germany

**Keywords:** Gender role, Gap, Female, Representation, Conference, Urology

## Abstract

**Purpose:**

Women are underrepresented at scientific conferences, decreasing the visibility of female role models, which are vital for aspiring young female scientists. This investigation aimed to evaluate female representation at the German Society of Urology's (GSoU) annual meeting.

**Methods:**

The programs of the GSoU meeting of 2011, 2018, 2019 and the virtual conference in 2020 were retrospectively quantified by gender and categorized by chair or speaker, type, and topic of the session. Descriptive analysis was applied. Univariate and multivariate analyses were performed to identify gender inequity and variables influencing gender distribution. A *p* value of < 0.05 was considered significant.

**Results:**

A total of 2.504 chairs and speakers were invited to the GSoU meeting in 2018 and 2019. Female speakers or chairs were represented in 17.8%, indicating a gender gap of 64.7%. There were significant differences between session type, topic, and gender distribution for chairs and speakers. The topic surgical techniques were independent variables for both, underrepresented female chairs and speakers, respectively (*p* < 0.001). Vocational policy and plenary session were not represented by any female chair in 2011, 2018, and 2019. In comparison, the gender gap in 2011 was 74.2%, indicating a gap reduction of 1.2% per year. In a selected virtual program in 2020, the gender gap increased to 70.4%.

**Conclusion:**

There is still a significant discrepancy between gender representation at the GSoU annual meetings, and gender equity is currently not expected before 50 years. Future efforts should address the implementation of established guidelines for achieving gender equity at urological conferences.

**Supplementary Information:**

The online version contains supplementary material available at 10.1007/s00345-021-03777-4.

## Introduction

Female physicians are mostly underrepresented at scientific medical conferences [1, 2]. Underrepresentation occurs when the number of female physicians in visible positions is less than the proportion expected based on the number of female physicians overall, including women in training [2]. This gender inequity is not limited to specific countries or disciplines, and similar reports have been described worldwide [3].

The lack of female representation as role models and mentors has been identified as a crucial barrier to promoting in surgical specialities and academic internal medicine [3, 4]. Unfortunately, mostly female discrimination puts women’s careers at a disadvantage and not the lack of appropriately skilled women [5]. Female representation at conferences is an essential facet of gender equity [2]. The numerical representation within an academic context may substantially influence perceptions of the climate. Thus, outnumbered female representation intensifies negatives outcomes for women [6]. Cochran et al. [4] identified the most significant barriers for women in academic departments of surgery are based on women's experiences. Academic conferences represent the gateway to an academic career and display the norm of a discipline [6] and, therefore, have been identified to reflect gender equity of a profession [2]. Yet, the gender gap has not been investigated in urological conferences.

The current investigation aimed to evaluate the gender imbalance of the GSoU annual conference and to provide future strategies to improve the urological field's attractiveness for talented women with alternative career options with more favourable environments [4].

## Materials and methods

The GSoU annual meeting program from 2011, 2018, and 2019 have been retrospectively reviewed to present the status quo and the change of female representation over time. Session type and topic were recorded and stratified by speaker and chair. For chairs, the academic position, title, and affiliation were analysed. Gender distribution is presented in absolute or relative numbers as appropriate. Univariate and multivariate analyses using a 95% confidence interval has been performed to identify variables influencing gender distribution. The variables gender, year, type, topic, title, affiliation, and academic position were included in this analysis. Finally, a pooled analysis of 2018 and 2019 was performed. A *p* value of < 0.05 was considered significant. Statistics have been performed with SPSS Version 26 (IBM, Armonk, United States).

Due to the COVID pandemic, the 2020 conference was virtual and a separate analysis of the "Best-Of-2020" program was conducted.

## Results

### Annual meeting 2011

A total of only 29 (9.0%) female chairs and 109 (14.6%) speakers were invited in 2011, indicating a gender gap of 74.2%.

There were significant differences between chairs’ gender and session type (*p* = 0.020) and between speakers’ gender and topic (*p* < 0.001) (Online Resource 1). Several session topics and types were not represented by any female chair and/or speaker, particularly plenary session and vocational policies (Online Resource 1 and 2).

### Annual meeting 2018 and 2019

A total of 2504 chairs and speakers have been invited in 2018 and 2019. Of these, 196 (15.8%) female chairs and 249 (19.8%) female speakers have been invited, indicating a gender gap of 64.7%. The estimated gap reduction within the last nine years was accordingly appr. 1.2% per year.

**Fig. 1:**
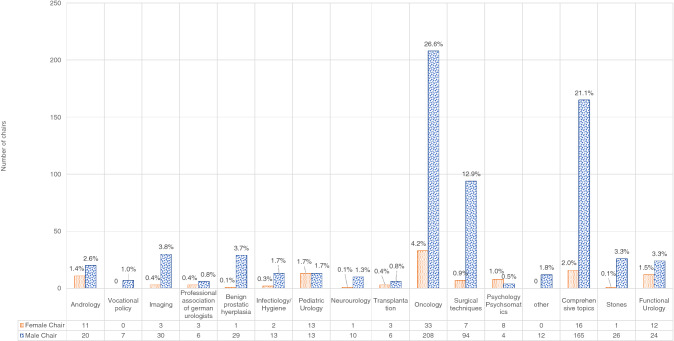
Chairs distributed by topic and gender in absolute numbers and percentages; cumulated for 2018 and 2019

There were significant differences between session topic (*p* < 0.001, Fig. 1, Online Resource 1 and 3), type (*p* = 0.013 and *p* < 0.001, Online Resource 2), and relative gender representation for chairs and speakers in 2018 and 2019, respectively.

No female chair represented vocational policy in both years, and the program committee invited only two female speakers (0.6%) in 2019.

There was no female chair invited for the plenary session in both years. Besides, only one female speaker was invited for the plenary session in 2019, considering both years.

In 2019, the topics benign prostatic enlargement, others and stones were moderated solely by men.

Female underrepresentation was also present, but not limited to, for speaker and chairs in the topic surgical techniques. Women more represented psychology and psychosomatic and pediatric urology. In both years, there was no male speaker for the session topic psychology and psychosomatic.

Oncologic topics, including any session topics with oncological subject, were significantly more often addressed by male than female speaker [*n* = 160 (9.3%) vs. *n* = 568 (33.0%), (*p* = 0.013)].

Regarding chairs, there were significant differences between gender distribution and academic position (*p* < 0.001), title (*p* < 0.001) and affiliation (*p* < 0.021) (Online Resource 4–6). The most common academic position of female chairs was an attending physician [n = 46 (5.9%)] in contrast to male chairs, which were most commonly chief physicians [*n* = 389, 49.8%)]. Full professorship was more often present in male chairs than in women [*n* = 53 (6.8%) vs. *n* = 464 (59.4%)].

### Multivariate analysis

Independent variables for underrepresented female chairs were the session topics imaging, benign prostatic enlargement, surgical techniques, comprehensive topics and stones. Furthermore, chief position and affiliation “other” was also independently associated with a male chair representation (Table 1).

**Table 1:** Logistic regression model of predictors for male chair representation

Variable/value	*p* value	Odds ratio	95% CI
**Session type**			
None			
**Session topic**			
Imaging	0.050*	4.34	0.99–18.87
Benign prostatic enlargement	0.006*	21.22	2.39–188.35
Surgical techniques	< 0.001*	7.91	2.55–24.56
Comprehensive topics	< 0.001*	8.22	2.85–23.71
Stones	0.012*	16.63	1.83–150.70
**Medical position**			
Chief physician	0.002*	4.78	1.76–12.99
**Affiliation**			
Other	0.028*	9.18	1.27–66.16
**Title**			
Doctor	0.019*	0.42	0.21–0.87

*Sig. *p* < 0.05; Affiliation “other” is defined by affiliation other than university hospital, municipal hospital or medical practice (e.g., lawyer)

Independent variables for underrepresented female speakers were benign prostatic enlargement and surgical techniques. Importantly, the session topic pediatric urology was independently correlated with an underrepresented male speaker. Female speaker underrepresentation was independently associated with the session types of plenary session, academic forum, academic expert session, and forum (Online Resource 7).

### Virtual conference due to COVID pandemic in 2020

A total of two (9.5%) female chairs and nine (14.8%) speakers were invited in 2020, representing a gender gap of 70.4%.

The session topics surgical techniques, robotics and laparoscopy, pension scheme for doctors in training, interventional benign prostatic enlargement, and localized and metastatic renal cell carcinoma were not represented by any female speaker.

## Discussion

Academic conferences are of major importance since they represent the gateway to an academic career and display a discipline's norm [6]. Therefore, female academic physicians' representation at academic conferences is a substantial landmark of gender equity [6, 7]. The greater the representation of women at conferences relatively to men, the more they feel less likely to perceive sexism and behave in a masculine manner [6]. Importantly, women who sense a meeting as sexist express increased intention to exit academic careers [6].

The current investigation identified a pooled gender gap of 64.7% at the GSoU annual meeting, with a total percentage of women of 17.8%. Considering the virtual meeting due to COVID pandemic with a highly selected program, the gender gap increased to 70.4%, with a total female representation of only 13.4%. The total number of urologists working in a hospital or ambulatory in 2019 was 5.932. Of these, 1.107 (18.7%) urologists were female [8]. In comparison to other disciplines, urology is still the discipline with the lowest female representation in Germany, and even behind the surgical disciplines (21.3%) [8]. Nevertheless, considering the total number of female clinically active urologists in Germany, female representation appears adequate since we have 18.7% female urologists and a representation of female urologists at the conference of 17.8%.

Still, some critical issues are not taken into consideration. The number of new certifications for medical specialists in urology in Germany in 2019 was 250, and of these, 38.8% were women [8]. Implying a trend for a significant increase of female urologists. Importantly, unconsidered is still the number of female physicians in urology training and official numbers are lacking for this article. Looking at the number of students in medicine, women already outnumber their male colleagues. In the year 2019, 62.5% of German medical students were female [9]. Contrary, female representation is decreasing rapidly in prestigious medical positions. The number of female chief urological physicians is only 18 (4.3%) in comparison to the total number of 414 chief physicians in urology in 2019. The number of self-employed women in doctor’s offices is only 291 (10.7%) from a total of 2.722 urological practices in Germany [8].

In comparison, in 2011, the total number of clinically active female urologists was 645/5.098 (12.7%) [10].

The number of female chief urological physicians was 10 (2.8%), and the number of self-employed women in doctor’s offices was 216 (7.9%). The annual meeting of the German Society of Urology in 2011 was represented by 12.9% women, thus, reflected the amount of female urologist at that time. This is consistent with the analysis of the years 2018 and 2019, representing an adequate increase of female representation in accordance with the corresponding number of female urologists.

In summary, the estimated increase of female representation at the GSoU annual meeting within eight years was approximately 5%. Considering a linear increase of female representation at the urological congress, gender equity will be achieved earliest in about 54 years. Concerning female chief urological physicians, there was an increase of 1.5% women, thus, gender equity is expected not before the next 240 years. Considering self-employed women in doctor’s offices there was an increase of 2.8%, reaching estimated gender equity not before 112 years.

However, considering a proportional increase of female representation according to the number of female urologists, as observed between 2011 and 2019, we will achieve gender equity earlier. Although, in our opinion, this trend must be proactively supported by the highest departmental and institutional levels to be accomplished.

An analysis of the session topics and types showed only one female speaker at the plenary session in both years and no female chair. This finding was confirmed as an independent predictor for underrepresented women. Furthermore, the session topic surgical techniques were independently correlated with female underrepresentation in both, female speakers and chairs. Contrary, female associated topics, i.e., *soft science*, such as pediatric urology or psychology and psychosomatic, were more frequently presented by women than men in absolute and relative numbers.

The lack of female plenary speakers is not only a urological phenomenon; it occurs in surgical conferences in up to 42.9% [1].

A predictor for female representation has been identified by the inclusion of women in the conference’s organization committee [1, 11]. In 2018 and 2019, the GSoU program committee consisted of only 1/16 (6.25%) and 2/19 (10.5%) women, respectively.

Zaza et al. [11] also reported, that women were less likely to present a clinical topic, technical skill, or moderate a scientific presentation. Contrary, they were more likely to give awards, introductions, keynote lectures, and soft science [11], which aligns with the current results.

Importantly, no female chair was represented in vocational policies; only two (1.1%) female speakers were invited in 2019, and no female speaker in 2018.

Furthermore, there were relatively more male chairs with a full professorship and affiliated to municipal hospitals in the current trial since they also possess more frequently than women a chief position and are more encouraged to receive full professorship.

This fact raises the question of the reason for these discrepancies. Frequently the pipeline problem is mentioned as causality [5]. The pipeline is an advanced explanation that suggests that gender inequality will decline once there are sufficient numbers of qualified numbers of women [5]. However, this explanation has been often identified as a persistent way of discrimination since various data have demonstrated that inequity often persists even if the number of women has increased [5]. For example, an American investigation on academia revealed that female associate professors constituted 10.9% but only 7.2% of full professors. On the contrary, male associate professors constituted 16.4%, but 28.1% were full professors, thus proving otherwise [5].

We are currently facing a significant gender gap in urology and must honestly ask ourselves for the reasons. Is it possible that we are still performing, even if unconsciously, female discrimination that impedes talented women from growing in higher senior positions and being present at urological conferences? The current trends are indicating an enormous change for the traditional, male-dominated discipline of urology. This is accompanied by various future challenges, which are included but are not limited to female representation in scientific conferences.

This investigation aimed to identify our discipline's current hard facts to call attention to the mandatory need for changes. Importantly, this is not a national problem; neither is it limited by boundaries, countries, or specific disciplines [6]. It is our collective burden.

We face a future of female urologists who might feel inadequate and even turn their backs on urology because of the lack of female representation, female role models, the lack of appropriate promotion and future perspective in a male-dominated discipline. Cochran et al. [4] identified the most significant barrier for female surgeons were external, i.e., female surgeons experiencing negative comments about their sex.

We acknowledge that many female urologists not seeking an academic urological career, and gender are not generally undermining a urological career. However, compared to men, female academic surgeons are less likely to marry and more likely to delay or forgo childbearing significantly [4]. And those having children have reported that this decision has come at the cost of their career [4]. Furthermore, women still earn less money than their male colleagues [12]. Whereas men are generally judged by their potential women’s evaluations, on the other hand, depending on their performances [13]. There are significant differences in wording and terminology even for recommendation letters on surgical residency, which may influence the selection process for female surgeons [14].

Even in this investigation, female physicians may decline participation for fear of reprisals. The expression of voice has been demonstrating empowering women because it can alter group norms [6]. However, it might be associated with negative consequences, and the costs are hostility, dislike, and even ostracism [6].

This environment of fear is confirmed in literature since it has become difficult to recognize and talk about gender gaps in the “post-equal opportunities” social world [15]. Gender inequity is tended to be originating from women themselves rather than from the environment. The popular solutions are individual support by, e.g., mentoring, rather than changing dominant cultures and practices that disadvantage women in the first place [15]. Unfortunately, even women may contribute to further gender inequity since they might not want their female identity made salient [15]. They might even feel encouraged to act in a male manner to access the discipline and its rewards [15].

We must ask ourselves where we want to see our discipline in the future. Is it an adequate environment where women do not have female role models and fear reprisal when expressing facts or concerns about gender bias? Gender inequity is everyone’s problem and not only for women or feminists [15].

Regarding academic conferences, simple guidelines have already been established to achieve gender balance. Martin et al. [16] published ten simple rules that include collecting data, developing a speaker policy, visibility of the policy, establishing a balanced and informed program committee, reporting data, building databases, responding to resistance, supporting women, and being family-friendly and taking the pledge. A key role is developing a speaker policy that describes the program committee's goals for its members and audience. The program committee must be familiar with these rules and be diverse and gender-balanced to succeed. Furthermore, it should be considered to fill the gap specifically in the male-dominated topics and session types to stop the female surgeons' association to “soft topics”.

Nevertheless, women themselves must contribute to this change as well. Women should take the pledge [16] if they are invited to academic conferences. As a common fact, a small number of studies identified women as being less likely to ask questions, women are more likely to decline invitations, and more likely to seek out shorter than more extensive talks [17].

Finally, the abolition of antiquated perceptions, development, and integration of female surgeons to the academic urological field must be actively supported and provided by the highest departmental and institutional levels [4] to ensure substantial change for our future female academic and non-academic surgeons.

We acknowledge the limitation of this investigation. Statistical data of female urologists in training are not available, limiting the exact evaluation of female representation. Analysis of the virtual conference and topics with a low number of female representations may be associated with a probability of a type II error since the total number of women was low.

## Conclusions

The GSoU's annual meeting is still facing a major gender gap. This implies a lack of female role models to encourage young talented women in the urology discipline. This underrepresentation may be associated with the perception of sexism and even the intention to exit academic careers [6]. To achieve gender equity, men and women must proactively take responsibility and hereby change our female future in urology.

Academic conferences are an essential gateway for an academic career. Simple rules may be followed to achieve gender balance for future meetings. One of the most critical steps is the inclusion of women in the program committee.

Finally, female urologists must be actively supported by programs [18] and provided by the highest departmental and institutional levels to herald a permanent change [4]. Leaders in academic medicine must hold people responsible, regardless of rank or position [18]. They must ensure an environment where women feel comfortable to express confidentially sexism accompanied by adequate measurements for unappropriated behaviors [18].

## Supplementary Information

Below is the link to the electronic supplementary material.

Supplementary file1 (DOCX 24 KB)

Supplementary file2 (DOCX 21 KB)

Supplementary file3 (DOCX 29 KB)

Supplementary file4 (DOCX 15 KB)

Supplementary file5 (DOCX 15 KB)

Supplementary file6 (DOCX 15 KB)

Supplementary file7 (DOCX 14 KB)

## Data Availability

Research data are not shared.

## References

[1] Gerull KM, Wahba BM, Goldin LM (2020). Representation of women in speaking roles at surgical conferences. Am J Surg.

[2] Ruzycki SM, Fletcher S, Earp M, Bharwani A, Lithgow KC (2019). Trends in the proportion of female speakers at Medical Conferences in the United States and in Canada, 2007 to 2017. JAMA Netw Open.

[3] Mascarenhas A, Moore JE, Tricco AC (2017). Perceptions and experiences of a gender gap at a Canadian research institute and potential strategies to mitigate this gap: a sequential mixed-methods study. CMAJ Open.

[4] Cochran A, Hauschild T, Elder WB, Neumayer LA, Brasel KJ, Crandall ML (2013). Perceived gender-based barriers to careers in academic surgery. Am J Surg.

[5] Monroe KR, Chiu WF (2010). Gender Equality in the Academy: The Pipeline Problem. PS: Polit Sci Polit.

[6] Biggs J, Hawley PH, Biernat M (2017). The academic conference as a chilly climate for women: effects of gender representation on experiences of sexism, coping responses, and career intentions. Sex Roles.

[7] Hoy K (2017). Gender imbalance at brain stimulation conferences: we have a problem and it is everyone's Problem. Brain Stimul.

[8] German Medical A. Ärztestatistik zum 31. Dezember 2019. [PDF]. 2019; https://www.bundesaerztekammer.de/fileadmin/user_upload/downloads/pdf-Ordner/Statistik2019/Stat19AbbTab.pdf. Accessed 02 Nov 2020

[9] Bundesamt S. Studierende insgesamt und Studierende Deutsche im Studienfach Medizin (Allgemein-Medizin) nach Geschlecht. 2019; https://www.destatis.de/DE/Themen/Gesellschaft-Umwelt/Bildung-Forschung-Kultur/Hochschulen/Tabellen/lrbil05.html. Accessed 03 Nov 2020

[10] Ärztestatistik zum 31. Dezember 2011. 2011. https://www.bundesaerztekammer.de/fileadmin/user_upload/downloads/Stat11Abbildungsteil1.pdf. Accessed 25 Nov 2020

[11] Zaza N, Ofshteyn A, Martinez-Quinones P, Sakran J, Stein SL (2020). Gender equity at surgical conferences: quantity and quality. J Surg Res.

[12] Longo P, Straehley CJ (2008). Whack! I've hit the glass ceiling! Women's efforts to gain status in surgery. Gend Med.

[13] Jacobs T (2019) Men are judged based on their potential; women are judged based on their past performance. https://psmag.com/economics/men-are-judged-based-on-their-potential-women-are-judged-based-on-their-past-performance

[14] Turrentine FE, Dreisbach CN, St Ivany AR, Hanks JB, Schroen AT (2019). Influence of gender on surgical residency applicants' recommendation letters. J Am Coll Surg.

[15] Riley S, Frith H, Archer L, Veseley L (2006). Institutional sexism in academia. The Psychologist.

[16] Martin JL (2014). Ten simple rules to achieve conference speaker gender balance. PLoS Comput Biol.

[17] Carter AJ, Croft A, Lukas D, Sandstrom GM (2018). Women's visibility in academic seminars: women ask fewer questions than men. PLoS ONE.

[18] Brown NJ (2020). Promoting the success of women and minority physician-scientists in academic medicine: a dean's perspective. J Clin Invest.

