# Supplementary material for: Gender gap at a large European urological congress: still at the beginning
Source: World J Urol. 2021 Jul 4;40(1):257–62. doi: 10.1007/s00345-021-03777-4 (PMC8813805; doi:10.1007/s00345-021-03777-4)
Supplement: Supplementary file 1 — Supplementary file1 (DOCX 24 KB) [file 345_2021_3777_MOESM1_ESM.docx]

Online Resource 1 Table Relative distribution of chairs and speaker according to the session topic in comparison between gender and year

|  | **Chair** | |  | **Speaker** | | |
| --- | --- | --- | --- | --- | --- | --- |
| **Year** | **2011** | **2018** | **2019** | **2011** | **2018** | **2019** |
| **p-value** | 0.087 | < 0.001* | < 0.001* | < 0.001* | < 0.001* | < 0.001* |
| **Session topic** |  |  |  |  |  |  |
| **Andrology, *n (%)*** |  |  |  |  |  |  |
| all gender | 16 (5.0) | 12 (3.3) | 19 (4.6) | 28 (3.7) | 30 (3.4) | 33 (3.9) |
| women | 4 (1.2) | 4 (1.1) | 7 (1.7) | 5 (0.7) | 7 (0.8) | 8 (09) |
| men | 12 (3.7) | 8 (2.2) | 12 (2.9) | 23 (3.1) | 23 (2.6) | 25 (3.0) |
| **Vocational policy, n *(%)*** |  |  |  |  |  |  |
| all gender | 9 (2.8) | 4 (1.1) | 3 (0.7) | 17 (2.3) | 10 (1.1) | 6 (0.7) |
| women | *0* | *0* | *0* | 3 (0.4) | *0* | 2 (0.2) |
| men | 9 (2.8) | 4 (1.1) | 3 (0.7) | 14 (1.9) | 10 (1.1) | 4 (0.5) |
| **Imaging, n *(%)*** |  |  |  |  |  |  |
| all gender | 4 (1.2) | 16 (4.4) | 17 (4.1) | 7 (0.9) | 22 (2.5) | 30 (3.6) |
| women | *0* | 1 (0.3) | 2 (0.5) | *0* | 1 (0.1) | 6 (0.7) |
| men | 4 (1.2) | 15 (4.1) | 15 (3.6) | 7 (0.9) | 21 (2.4) | 24 (2.8) |
| **Benign prostatic hyperplasia, *n (%)*** |  |  |  |  |  |  |
| all gender | 8 (2.5) | 12 (3.3) | 18 (4.3) | 24 (3.2) | 30 (3.4) | 33 (3.9) |
| women | *0* | 1 (0.3) | *0* | *0* | 2 (0.2) | 3 (0.4) |
| men | 8 (2.5) | 11 (3.0) | 18 (4.3) | 24 (3.2) | 28 (3.2) | 30 (3.6) |
| **Professional association of german urologist, *n (%)*** |  |  |  |  |  |  |
| all gender | NA | 3 (0.8) | 6 (1.4) | NA | 12 (1.4) | 10 (1.2) |
| women | NA | 1 (0.3) | 2 (0.5) | NA | 1 (0.1) | 1 (0.1) |
| men | NA | 2 (0.5) | 4 (1.0) | NA | 11 (1.3) | 9 (1.1) |
| **Infectiology and hygiene, *n (%)*** |  |  |  |  |  |  |
| all gender | 6 (1.9) | 9 (2.5) | 6 (1.4) | 12 (1.6) | 22 (2.5) | 15 (1.8) |
| women | *0* | 1 (0.3) | 1 (0.2) | 0 | 5 (0.6) | 4 (0.5) |
| men | 6 (1.9) | 8 (2.2) | 5 (1.2) | 12 (1.6) | 17 (1.9) | 11 (1.3) |
| **Pediatric urology, *n (%)*** |  |  |  |  |  |  |
| all gender | 11 (3.4) | 16 (4.4) | 10 (2.4) | 25 (3.3) | 23 (3.6) | 18 (2.1) |
| women | *0* | *8 (2.2)* | 5 (1.2) | 11 (1.5) | 13 (1.5) | 13 (1.5) |
| men | 11 (3.4) | *8 (2.2)* | 5 (1.2) | 14 (1.9) | 19 (2.2) | 5 (0.6) |
| **Neurourology, *n (%)*** |  |  |  |  |  |  |
| all gender | NA | 8 (2.2) | 3 (0.7) | NA | 23 (2.6) | 11 (1.3) |
| women | NA | *0* | 1 (0.2) | NA | 8 (0.9) | 2 (0.2) |
| men | NA | 8 (2.2) | 2 (0.5) | NA | 15 (1.7) | 9 (1.1) |
| **Transplantation, *n (%)*** |  |  |  |  |  |  |
| all gender | 8 (2.5) | 6 (1.6) | 3 (0.7) | 18 (2.4) | 16 (1.8) | 6 (0.7) |
| women | *0* | 2 (0.5) | 1 (0.2) | 4 (0.5) | 6 (0.7) | 1 (0.1) |
| men | 8 (2.5) | 4 (1.1) | 2 (0.5) | 14 (1.9) | 10 (1.1) | 5 (0.6) |
| **Oncology, *n (%)*** |  |  |  |  |  |  |
| all gender | 101 (31.4) | 108 (29.5) | 133 (32.0) | 267 (35.7) | 291 (33.1) | 327 (38.7) |
| women | 7 (2.2) | 13 (3.6) | 20 (4.8) | 42 (5.6) | 57 (6.5) | *89 (10.5)* |
| men | 94 (29.2) | 95 (26.0) | 113 (27.2) | 225 (30.1) | 234 (26.7) | *238 (28.2)* |
| **Surgical technique, *n (%)*** |  |  |  |  |  |  |
| all gender | 57 (17.7) | 55 (15.0) | 46 (11.1) | 125 (126.7) | 144 (16.4) | 74 (8.8) |
| women | 4 (1.2) | 4 (1.1) | 3 (0.7) | *6 (0.8)* | *7 (0.8)* | 9 (1.1) |
| men | 53 (16.5) | 51 (13.9) | 43 (10.4) | *119 (15.9)* | *137 (15.6)* | 65 (7.7) |
| **Psychology and Psychosomatic, *n (%)*** |  |  |  |  |  |  |
| all gender | 2 (0.6) | 5 (1.4) | 7 (1.7) | 3 (0.4) | 3 (0.3) | 3 (0.4) |
| women | *0* | 4 (1.1) | 4 (1.0) | 1 (0.1) | 3 (0.3) | 3 (0.4) |
| men | 2 (0.6) | 1 (0.3) | 3 (0.7) | 2 (0.3) | *0* | *0* |
| **Other, *n (%)*** |  |  |  |  |  |  |
| all gender | 30 (9.3) | NA | 12 (2.9) | 68 (9.1) | NA | 23 (2.7) |
| women | 3 (0.9) | NA | *0* | 7 (0.9) | NA | 3 (0.4) |
| men | 27 (8.4) | NA | 12 (2.9) | 61 (8.2) | NA | 20 (2.4) |
| **Comprehensive topics, *n (%)*** |  |  |  |  |  |  |
| all gender | 31 (9.6) | 85 (23.2) | 96 (23.1) | 55 (7.4) | 181 (20.6) | 182 (21.5) |
| women | 4 (1.2) | *7 (1.9)* | *9 (2.2)* | 11 (1.5) | 24 (2.7) | 30 (3.6) |
| men | 27 (8.4) | *78 (21.3)* | *87 (21.0)* | 44 (5.9) | 157 (17.9) | 152 (18.0) |
| **Stones, *n (%)*** |  |  |  |  |  |  |
| all gender | 17 (5.3) | 15 (4.1) | 12 (2.9) | 46 (6.2) | 36 (4.1) | 32 (3.8) |
| women | 1 (0.3) | 1 (0.3) | *0* | 8 (1.1) | 5 (0.6) | 2 (0.2) |
| men | 16 (5.0) | 14 (3.8) | 12 (2.9) | 38 (5.1) | 31 (3.5) | 30 (3.6) |
| **Functional urology, *n (%)*** |  |  |  |  |  |  |
| all gender | 22 (6.8) | 12 (3.3) | 24 (5.8) | 52 (7.0) | 26 (3.0) | 42 (5.0) |
| women | 6 (1.9) | 5 (1.4) | 7 (1.7) | 11 (1.5) | 5 (0.6) | 11 (1.3) |
| men | 16 (5.0) | 7 (1.9) | 17 (4.1) | 41 (5.5) | 21 (2.4) | 31 (3.7) |
| NA not applicable, *Sig. p < 0.005 | | | | | | |
