# Supplementary material for: Gender gap at a large European urological congress: still at the beginning
Source: World J Urol. 2021 Jul 4;40(1):257–62. doi: 10.1007/s00345-021-03777-4 (PMC8813805; doi:10.1007/s00345-021-03777-4)
Supplement: Supplementary file 2 — Supplementary file2 (DOCX 21 KB) [file 345_2021_3777_MOESM2_ESM.docx]

Online Resource 2 Table Relative distribution of chairs and speaker according to the session type in comparison between gender and year

|  | **Chair** | | | **Speaker** | | |
| --- | --- | --- | --- | --- | --- | --- |
| **Year** | **2011** | **2018** | **2019** | **2011** | **2018** | **2019** |
| **p-value** | 0.020* | 0.027* | 0.460 | 0.146 | <0.001* | <0.001* |
| **Session type** |  |  |  |  |  |  |
| **Forum, n (%)** |  |  |  |  |  |  |
| all gender | 60 (18.6) | 124 (33.9) | 163 (39.3) | 82 (11.0) | 303 (34.5) | 304 (36.0) |
| women | 3 (0.9) | 14 (3.8) | 24 (5.8) | 7 (6.4) | 36 (4.1) | 48 (5.7) |
| men | 57 (17.7) | 110 (30.1) | 139 (33.5) | 75 (11.8) | 267 (30.4) | 256 (30.3) |
| **Abstract / lecture session, n (%)** |  |  |  |  |  |  |
| all gender | 156 (48.4) | 126 (29.5) | 119 (28.7) | 466 (62.4) | 369 (42.0) | 341 (40.4) |
| women | 12 (3.7) | 18 (4.9) | 18 (4.3) | 81 (74.3) | 91 (10.4) | 105 (12.4) |
| men | 144 (44.7) | 108 (29.5) | 101 (24.3) | 385 (60.3) | 278 (31.7) | 236 (27.9) |
| **Academy expert session, n (%)** |  |  |  |  |  |  |
| all gender | 41 (12.7) | 47 (12.8) | 54 (13.) | 61 (8.2) | 76 (8.7) | 50 (5.9) |
| women | 8 (2.5) | 14 (3.8) | 13 (3.1) | 4 (3.7) | 6 (0.7) | 13 (1.5) |
| men | 33 (10.2) | 33 (9.0) | 41 (9.9) | 57 (8.9) | 70 (8.0) | 37 (4.4 |
| **Academy forum, n (%)** |  |  |  |  |  |  |
| all gender | 42 (13.0) | 48 (13.1) | 53 (12.8) | 103 (13.8) | 95 (10.8) | 87 (10.3) |
| women | 3 (0.9) | 5 (1.4) | 5 (1.2) | 12 (11.0) | 10 (1.1) | 14 (1.7) |
| men | 39 (12.1) | 43 (11.7) | 48 (11.6) | 91 (14.3) | 85 (9.7) | 73 (8.6) |
| **Seminar, n (%)** |  |  |  |  |  |  |
| all gender | NA | 12 (3.3) | 11 (2.7) | NA | 11 (1.3) | 14 (1.7) |
| women | NA | 1 (0.3) | 1 (0.2) | NA | 1 (0.1) | 1 (0.5) |
| men | NA | 11 (3.0) | 10 (2.4) | NA | 10 (1.1) | 13 (2.4) |
| **Plenary session, n (%)** |  |  |  |  |  |  |
| all gender | 9 (2.8) | 9 (2.5) | 6 (1.4) | 18 (2.4) | 21 (2.4) | 14 (1.7) |
| women | 0 | 0 | 0 | 2 (1.8) | 0 | 1 (0.1) |
| men | 9 (2.8) | 9 (2.5) | 6 (1.4) | 16 (2.5) | 21 (2.4) | 13 (1.5) |
| **Video session, n (%)** |  |  |  |  |  |  |
| all gender | 3 (0.9) | NA | 6 (1.4) | 5 (0.7) | NA | 15 (1.8) |
| women | 0 | NA | 1 (0.2) | 1 (0.9) | NA | 2 (0.2) |
| men | 3 (0.9) | NA | 5 (1.2) | 4 (0.6) | NA | 13 (1.5) |
| **Other, n (%)** |  |  |  |  |  |  |
| all gender | 9 (2.8) | NA | 3 (0.7) | 12 (1.6) | NA | 10 (1.2) |
| women | 3 (0.9) | NA | 0 | 2 (1.8) | NA | 0 |
| men | 6 (1.9) | NA | 3 (0.7) | 10 (1.6) | NA | 10 (1.2) |
| NA not applicable, *Sig. p < 0.005 | | | | | | |
