# Supplementary material for: Gender gap at a large European urological congress: still at the beginning
Source: World J Urol. 2021 Jul 4;40(1):257–62. doi: 10.1007/s00345-021-03777-4 (PMC8813805; doi:10.1007/s00345-021-03777-4)
Supplement: Supplementary file 3 — Supplementary file3 (DOCX 29 KB) [file 345_2021_3777_MOESM3_ESM.docx]

# Online Resource 3 Figure Speaker distributed by topic and gender in absolute numbers and percentages, cumulated for 2018 and 2019
