# Supplementary material for: Gender gap at a large European urological congress: still at the beginning
Source: World J Urol. 2021 Jul 4;40(1):257–62. doi: 10.1007/s00345-021-03777-4 (PMC8813805; doi:10.1007/s00345-021-03777-4)
Supplement: Supplementary file 4 — Supplementary file4 (DOCX 15 KB) [file 345_2021_3777_MOESM4_ESM.docx]

Online Resource 4 Table Medical position of chairs in comparison between gender and year

| **Year**  **Position p-value** | **2018** | **2019** | **2018 – 2019** |
| --- | --- | --- | --- |
|  | <0.001* | <0.001* | <0.001* |
| **Resident, n (%)** |  |  |  |
| all gender | 8 (2.2) | 1 (0.2) | 9 (1.2) |
| women | 5 (1.4) | 1 (0.2) | 6 (0.8) |
| men | 3 (0.8) | 0 | 3 (0.4) |
| **Chief physician, n (%)** |  |  |  |
| all gender | 203 (55.5) | 219 (52.8) | 422 (54.0) |
| women | 13 (3.6) | 20 (4.8) | 33 (4.2) |
| men | 190 (51.9) | 199 (48.0) | 389 (49.8) |
| **Medical specialist, n (%)** |  |  |  |
| all gender | 0 | 5 (1.2) | 5 (0.6) |
| women | 0 | 4 (1.0) | 4 (0.5) |
| men | 0 | 1 (0.2) | 1 (0.1) |
| **Practitioner, n (%)** |  |  |  |
| all gender | 41 (11.2) | 41 (9.9) | 82 (10.5) |
| women | 6 (1.6) | 6 (1.4) | 12 (1.5) |
| men | 35 (9.6) | 35 (8.4) | 70 (9.0) |
| **Attending physician, n (%)** |  |  |  |
| all gender | 85 (23.2) | 117 (28.2) | 202 (25.9) |
| women | 20 (1.4) | 26 (6.3) | 46 (5.9) |
| men | 65 (0.8) | 91 (21.9) | 156 (20.0) |
| **Other, n (%)** |  |  |  |
| all gender | 29 (7.9) | 32 (7.7) | 61 (7.8) |
| women | 8 (2.2) | 5 (1.2) | 13 (1.7) |
| men | 21 (5.7) | 27 (6.5) | 48 (6.1) |
| NA not applicable; *Sig. p < 0.05 | | | |
