# Supplementary material for: Gender gap at a large European urological congress: still at the beginning
Source: World J Urol. 2021 Jul 4;40(1):257–62. doi: 10.1007/s00345-021-03777-4 (PMC8813805; doi:10.1007/s00345-021-03777-4)
Supplement: Supplementary file 5 — Supplementary file5 (DOCX 15 KB) [file 345_2021_3777_MOESM5_ESM.docx]

Online Resource 5 Table Distribution of chair title in comparison between gender and year

| **Year**  **Title p-value** | **2018** | **2019** | **2018 – 2019** |
| --- | --- | --- | --- |
|  | <0.001* | 0.043* | <0.001* |
| **No title, n (%)** |  |  |  |
| all gender | 6 (1.6) | 4 (1.0) | 10 (1.3) |
| women | 2 (0.5) | 2 (0.5) | 4 (0.5) |
| men | 4 (1.1) | 2 (0.5) | 6 (0.8) |
| **Doctor degree, n (%)** |  |  |  |
| all gender | 68 (18.6) | 66 (15.9) | 134 (17.2) |
| women | 21 (5.7) | 14 (3.4) | 35 (4.5) |
| men | 47 (12.8) | 52 (12.5) | 99 (12.7) |
| **Associate Professor, n (%)** |  |  |  |
| all gender | 41 (11.2) | 79 (19.0) | 120 (15.4) |
| women | 8 (2.2) | 14 (3.4) | 22 (2.8) |
| men | 33 (9.0) | 65 (15.7) | 98 (12.5) |
| **Full Professor, n (%)** |  |  |  |
| all gender | 251 (68.6) | 266 (64.1) | 517 (66.2) |
| women | 21 (5.7) | 32 (7.7) | 53 (6.8) |
| men | 230 (62.8) | 234 (56.4) | 464 (59.4) |
| NA not applicable, *Sig. p < 0.05 | | | |
