# Supplementary material for: Gender gap at a large European urological congress: still at the beginning
Source: World J Urol. 2021 Jul 4;40(1):257–62. doi: 10.1007/s00345-021-03777-4 (PMC8813805; doi:10.1007/s00345-021-03777-4)
Supplement: Supplementary file 6 — Supplementary file6 (DOCX 15 KB) [file 345_2021_3777_MOESM6_ESM.docx]

Online Resource 6 Table Distribution of chair affiliation in comparison between gender and year

| **Year**  **Affiliation p-value** | **2018** | **2019** | **2018 – 2019** |
| --- | --- | --- | --- |
|  | 0.652 | 0.018* | 0.021* |
| **University, n (%)** |  |  |  |
| all gender | 190 (51.9) | 204 (49.2) | 394 (50.4) |
| women | 31 (8.5) | 41 (9.9) | 72 (9.2) |
| men | 159 (43.4) | 163 (39.9) | 322 (41.2) |
| **Municipal hospital, n (%)** |  |  |  |
| all gender | 117 (32.0) | 160 (38.6) | 277 (35.5) |
| women | 13 (3.6) | 15 (3.6) | 28 (3.6) |
| men | 104 (28.4) | 145 (34.9) | 249 (31.9) |
| **Medical practice, n (%)** |  |  |  |
| all gender | 44 (12.0) | 40 (9.6) | 84 (10.8) |
| women | 6 (1.6) | 6 (1.4) | 12 (1.5) |
| men | 38 (10.4) | 34 (8.2) | 72 (9.2) |
| **Other, n (%)** |  |  |  |
| all gender | 15 (4.1) | 11 (2.7) | 26 (3.3) |
| women | 2 (0.5) | 0 | 2 (0.3) |
| men | 13 (3.6) | 11 (2.7) | 24 (3.1) |
| NA not applicable; Sig. p < 0.005; Affiliation “other” is defined by affiliation other than university hospital, municipal hospital or medical practice (e.g. lawyer) | | | |
