# Supplementary material for: Gender gap at a large European urological congress: still at the beginning
Source: World J Urol. 2021 Jul 4;40(1):257–62. doi: 10.1007/s00345-021-03777-4 (PMC8813805; doi:10.1007/s00345-021-03777-4)
Supplement: Supplementary file 7 — Supplementary file7 (DOCX 14 KB) [file 345_2021_3777_MOESM7_ESM.docx]

Online Resource 7 Table Logistic regression model of predictors for male speaker representation

| **Value**  **Variable** | **p-value** | **Odds ratio** | **95 % CI** |
| --- | --- | --- | --- |
| **Session Type** | | | |
| Academy expert session | 0.024* | 2.16 | 1.11 – 4.20 |
| Academy forum | < 0.001* | 4.00 | 2.26 – 7.07 |
| Forum | < 0.001* | 2.49 | 1.80 – 3.44 |
| Plenary session | 0.014* | 12.75 | 1.68 – 96.69 |
| **Session topic** | | | |
| Benign prostatic enlargement | 0.002* | 5.71 | 1.88 – 17.36 |
| Pediatric urology | 0.001* | 0.26 | 0.12 – 0.59 |
| Surgical technique | < 0.001* | 4.10 | 1.86 – 9.04 |
| **Year** | | | |
| Year | < 0.001* | 0.65 | 0.50 – 0.84 |
| *Sig. p < 0.05 | | | |
